# Socioeconomic burden of hereditary angioedema: results from the hereditary angioedema burden of illness study in Europe

**DOI:** 10.1186/1750-1172-9-99

**Published:** 2014-07-04

**Authors:** Emel Aygören-Pürsün, Anette Bygum, Kathleen Beusterien, Emily Hautamaki, Zlatko Sisic, Suzanne Wait, Henrik B Boysen, Teresa Caballero

**Affiliations:** 1 Department for Children and Adolescents, Angioedema Centre, University Hospital Frankfurt, Goethe University, Theodor-Stern-Kai 7, 60596 Frankfurt, Germany; 2 HAE Centre Denmark, Department of Dermatology and Allergy Centre, Odense University Hospital, 5000 Odense C, Denmark; 3 Oxford Outcomes Inc., an ICON plc company, 7315 Wisconsin Ave, Suite 250 W, 20814 Bethesda, MD, USA; 4 ViroPharma, Chatsworth House, 29 Broadway, SL6 1LY Maidenhead, UK; 5 SHW Health Ltd, 40 Lena Gardens, W6 7PZ London, UK; 6 HAEi – International Patient Organization for C1 Inhibitor Deficiencies, Soenderholmvej 158, DK-8361 Bering, Hasselager, Denmark; 7 Allergy Department, Hospital La Paz Institute for Health Research (IdiPaz), Biomedical Research Network on Rare Diseases U754 (CIBERER), University Hospital La Paz, Paseo de la Castellana 261, 28046 Madrid, Spain

**Keywords:** Hereditary angioedema, Burden of illness, Resource utilization, Productivity

## Abstract

**Background:**

Hereditary angioedema (HAE) due to C1 inhibitor deficiency is a rare but serious and potentially life-threatening disease marked by spontaneous, recurrent attacks of swelling. The study objective was to characterize direct and indirect resource utilization associated with HAE from the patient perspective in Europe.

**Methods:**

The study was conducted in Spain, Germany, and Denmark to assess the real-world experience of HAE via a cross-sectional survey of HAE patients, including direct and indirect resource utilization during and between attacks for patients and their caregivers over the past 6 months. A regression model examined predictors of medical resource utilization.

**Results:**

Overall, 164 patients had an attack in the past 6 months and were included in the analysis. The most significant predictor of medical resource utilization was the severity of the last attack (OR 2.6; p < 0.001). Among patients who sought medical care during the last attack (23%), more than half utilized the emergency department. The last attack prevented patients from their normal activities an average of 4–12 hours. Patient and caregiver absenteeism increased with attack severity and frequency. Among patients who were working or in school (n = 120), 72 provided work/school absenteeism data, resulting in an estimated 20 days missing from work/school on average per year; 51% (n = 84) indicated that HAE has hindered their career/educational advancement.

**Conclusion:**

HAE poses a considerable burden on patients and their families in terms of direct medical costs and indirect costs related to lost productivity. This burden is substantial at the time of attacks and in between attacks.

## Introduction

Hereditary angioedema (HAE) is an autosomal dominant disorder caused by a deficiency of C1 inhibitor. It is a rare but serious and potentially life-threatening disease marked by spontaneous, recurrent attacks of swelling, typically in the gastrointestinal tract, extremities, face, larynx, and/or urogenitals [1-6]. The prevalence has been estimated to be around 1 in 50,000 [6,7]. Attacks vary unpredictably with respect to severity, frequency, and body site, and can be life-threatening due to risk of asphyxiation [4,8,9]. Symptoms often begin in early childhood and persist throughout patients’ lives, and an accurate diagnosis may be delayed for 10 years or more [2,5,6].

While there is no cure for HAE, existing treatment options have substantially reduced mortality [10]. Treatment strategies include treatment of acute attacks, pre-procedure attack prophylaxis, and long-term prophylaxis to minimize the frequency and severity of attacks [11-16]. The available treatment options at the time of the study varied between countries and mostly consisted of a plasma derived C1 inhibitor concentrate and a B2 bradykinin receptor antagonist for the treatment of acute attacks [14-16]. Long-term prophylaxis options have traditionally involved attenuated androgens and anti-fibrinolytic agents, although their approval status for HAE varies among European countries [14-17]. Recently, two additional treatment options became available in Europe: a recombinant C1 inhibitor for the treatment of acute attacks [18] and a nanofiltered C1 inhibitor [human] for the treatment, pre-procedure prevention, and long-term prevention of angioedema attacks in adults and adolescents with HAE [19].

Given the unpredictable nature of HAE and the severity of attacks, use of emergency medical care may be high. Based on a nationwide database in the US, there were 5040 emergency department visits for HAE in 2006–2007, 41% of which resulted in hospitalization [20]. Moreover, the findings from a survey of individuals with HAE in the US showed that HAE may have a substantial impact on work productivity and medical resource utilization [21]. However, such data on the socioeconomic consequences of HAE are lacking in Europe. Also, the use of caregivers for HAE has not been explored previously. Thus, the objective of this study was to characterize direct and indirect resource utilization associated with HAE from the patient perspective in Europe, focusing on Spain, Germany, and Denmark.

## Methods

The Hereditary Angioedema Burden of Illness Study in Europe (HAE-BOIS-Europe) was a cross-sectional study aimed at assessing the real-world patient experience of HAE in Spain, Germany, and Denmark. Patients at least 12 years of age who were diagnosed with HAE type I or type II were recruited from patient organizations and from clinical practice centers of excellence. In Spain, all members of the patient organization and a random sample of patients in the Spanish Registry Database (which includes patients from across the country) were invited; in Germany, all members of the patient organization and a random sample of patients from the clinical center database were invited; and in Denmark, all known patients with HAE in the country were invited between the clinical center and the patient organization.

Patients completed a web- or paper-based survey, depending on their preference. Data collection took place in May-December 2011. The study was reviewed and approved by institutional review boards per local requirements. Participants provided online informed consent (or written informed consent if completing the paper version of the survey) prior to taking the survey. For participants under the age of 18, parental consent was obtained in addition to the adolescent’s assent. The survey was developed based on consultation with HAE clinical experts and pilot-tested with patients in each country. Items on clinical characteristics, humanistic burden, and resource utilization were collected retrospectively. Patients were asked about their most recent attack, as well as about the impact of HAE between attacks over the last 6 months. The study was not designed to examine specific treatment outcomes; however, patients were asked to identify their current and previous HAE medications. The full methodology and humanistic burden data from this study are described elsewhere [22,23].

This manuscript reports the results related to direct medical resource utilization (medication use for acute attacks and prophylaxis, acute or emergency medical care for the last attack, routine care visits, and use of antianxiety or antidepressant medication) and indirect resource utilization (lost productivity and absenteeism from work/school and time lost from usual activities during attacks and between attacks for the patient and their caregivers). The impact of HAE on work/school productivity both during the recent attack and in between attacks over the past six months was measured on an 11-point numeric rating scale, consistent with other patient-reported measures of productivity [24]. The survey items and scoring are reported in Table 1. An annual rate of work/school absenteeism for HAE patients was estimated from the reported amount of time missed from work/school for the last attack, frequency of attacks, and the reported amount of time missed from work/school between attacks over the past six months (among patients with non-missing data). Caregivers were defined as “a volunteer (unpaid) helper because of HAE (e.g., to aid in your ability to attend your medical appointments or to assist you in your daily activities)”. The use of caregivers (including the amount of time they missed from work/school/leisure while assisting the HAE patient), both during the last attack and between attacks, was also collected.

**Table 1:** Survey items on direct and indirect resource utilization

**Concept**		**Item**	**Scoring**
Direct medical resource utilization over the past 6 months	“Treatment visit for last attack”	Please report the type of visit you had, to the best of your knowledge, for your most recent attack.	Endorsement of 0 or 1 item = low
		*Endorsement of at least one type of treatment visit = 1*	
	“Used medication for last attack”	Which prescription medication(s) were used for the treatment of your most recent attack? Please indicate the name/type of all medication prescribed by your doctor(s) you used for the most recent HAE attack.	Endorsement of 2 = medium
		*Endorsement of at least one type of medication = 1*	Endorsement of 3 or 4 = high
	“Had a routine care visit for HAE in the past 6 months”	In the past 6 months, how many times have you been to the hospital or clinic for routine care related to HAE? Please exclude visits related to HAE attacks.	
		*Endorsement of at least one visit = 1*	
	“Used anti-anxiety or anti-depressant medication”	Which of the following medications have you taken during the past 6 months (check all that apply)?	
		*Endorsement of “anti-anxiety medication” and/or “anti-depressants” = 1*	
Estimated absenteeism over one year	“Attacks per year”	HAE attacks may be mild, moderate, or severe. In the past year, approximately how often did you experience an HAE attack of any severity (please account for all attacks regardless of severity)?	1. Reported attack frequency extrapolated to estimate annual frequency.
		• *Less than once a month* (Approximately how many per year?___)	2. Annual frequency multiplied by amount of absenteeism for the last attack.
		• *At least once a month but less than once a week* (Approximately how many per month?___)	3. Added to twice the amount of absenteeism between attacks over the past 6 months.
		• *At least once a week* (Approximately how many per week?___)	
	“Absenteeism during the last attack”	During your most recent attack, how many hours or days did you miss from work and/or school because of problems associated with the HAE attack? Include hours you missed on sick days, times you arrived late, left early, etc., because of the HAE attack.	
	“Absenteeism between attacks over the past 6 months”	In the past six months, how many days did you miss from work and/or school because of problems associated with HAE in between attacks? Include hours you missed on sick days, times you arrived late, left early, etc., in between attacks.	
Attack severity	“Attack pain severity”	What was the worst pain you had during your most recent HAE attack? *0 = No pain; 1 = Mild; 2 = Moderate; 3 = Severe*	Average of pain and swelling scores
	“Attack swelling severity”	How would you describe the severity of swelling of your most recent attack?	
		• *1 = Mild (noticeable symptoms but they did not affect your daily activities)*	
		• *2 = Moderate (you wanted intervention for symptoms during the attack or your daily activities were affected)*	
		• *3 = Severe (treatment or intervention was required or you were unable to perform daily activities)*	

Analyses were descriptive, reporting means and frequencies, and differences among subgroups were explored using t-tests and chi-square tests for continuous and categorical variables, respectively. Two logistic regression models were developed to evaluate associations between potential explanatory variables and 1) having a treatment visit for the last attack, and 2) “six-month medical resource utilization”, as described in Table 1.

Potential explanatory variables included the severity of the last attack, duration of swelling of the last attack, attack frequency, country, age, gender, household income, having medication at home for self-administration during attacks, and use of medication for long-term prophylaxis (by type). The substantial differences in treatment patterns and healthcare system configurations in the three countries allowed for only minimal comparisons across countries.

## Results

Of the 186 HAE-BOIS-Europe participants, 164 (88%) experienced an attack in the past six months and formed the analysis sample, including 58 (35%) from Spain, 62 (38%) from Germany, and 44 (27%) from Denmark.

### Patients’ characteristics

The average age was 43 years and 100 (61%) were female. Patients were diagnosed with HAE an average of 12 years after symptom onset. Thirty-eight patients (23%) reported having HAE attacks at least once a week, 67 (41%) reported having attacks at least once a month but less than once a week, and 59 (36%) reported having attacks less than once a month. The average number of attacks per year (estimated) was 15, 52, and 29 in Spain, Germany, and Denmark, respectively. Fifty-four patients (33%) reported experiencing no pain or mild pain during the last HAE attack, 74 (45%) reported experiencing moderate pain, and 36 (22%) reported experiencing severe pain; this did not vary significantly by country.

### Direct resource utilization

#### *Treatment of the acute attack*

**Figure 1:**
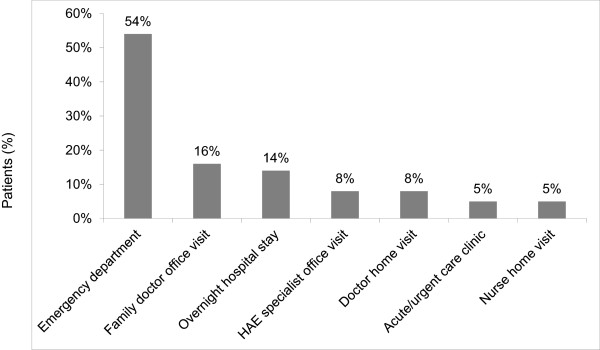
**Types of treatment visits for most recent HAE attack (N = 37 patients).** Note: Four patients had more than one type of treatment visit for the attack

Nearly all patients (n = 150; 91%) had medication at home to treat acute attacks. A total of 109 patients (66%) reported that they treated the attack with an HAE acute-specific medication, either plasma-derived C1 inhibitor and/or B2 bradykinin receptor antagonist. Even though the countries did not differ significantly with respect to the severity of the last attack, the use of HAE acute-specific medication for the last attack varied among the three countries [90% (n = 56) in Germany, 61% (n = 27) in Denmark, and 45% (n = 26) in Spain; p < 0.001]. This also varied by attack frequency; 100% (n = 38) of patients who experienced attacks at least once a week used an appropriate acute medication, compared with 70% (n = 47) of patients with attacks at least once a month but less than once a week, and 41% (n = 24) of patients with attacks less than once a month (p < 0.001). Among patients who experienced severe pain during their last attack, 83% (n = 30) used an appropriate acute medication, compared to 72% (n = 53) of patients with moderate pain, and 48% (n = 26) of patients with mild pain (p = 0.001). Overall, 22% (n = 36) of patients did not take any medication for the last attack.A total of 37 patients (23%) sought medical care from at least one source during the last attack, with the emergency department being the most often visited (n = 20; 54%); 5 of the patients (14%) were hospitalized at least overnight (Figure 1). Use of emergency departments for the last attack increased with increasing pain severity of the last attack [19% (n = 7) of patients with severe pain used an emergency department compared to 16% (n = 12) of patients with moderate pain and 2% (n = 1) of patients with mild or no pain; (p = 0.016)]. The severity of the last attack (as measured by pain and swelling) was also a strong predictor in the regression models of treatment visits for the last attack and of six-month medical resource utilization. Specifically, as attack severity increased by 1 point on a scale from 0 (least severe) to 3 (most severe), patients were 3.0 times more likely to have a treatment visit (p < 0.001) and 2.6 times more likely to have higher six-month medical resource utilization (p < 0.001). Also, for each increase in swelling duration of approximately 12 hours, patients were 1.5 times more likely to have a treatment visit (p = 0.008).

#### *Treatment in between attacks*

Over the past six months, 98 patients (60%) reported seeing a healthcare provider for HAE in between attacks. Use of long-term prophylaxis with either attenuated androgens or tranexamic acid was reported by 56 patients (34%), the majority of whom (n = 40; 71%) were taking attenuated androgens. Overall, 61 patients (37%) reported discontinuing one or more long-term prophylaxis medications in the past, primarily due to side effects or lack of efficacy. Among these, 38 (62%) discontinued attenuated androgens, 18 (30%) discontinued tranexamic acid, and 5 (8%) discontinued both tranexamic acid and attenuated androgens. In addition, 17 patients (10%) had used an anti-anxiety or antidepressant medication in the past six months.

### Indirect resource utilization

#### *Impact of the acute attack on productivity for patients and their caregivers*

**Figure 2:**
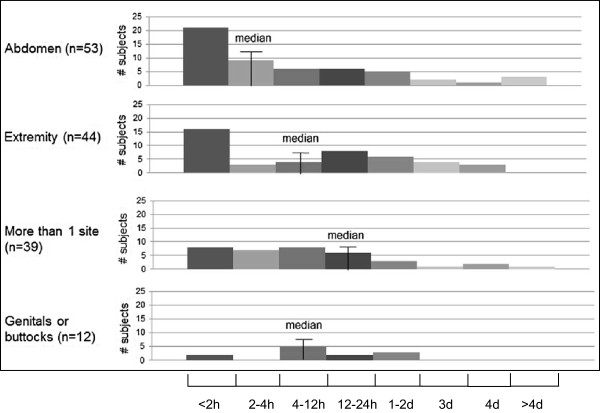
**Duration prevented from daily activities by attack site**^**a**^**. **^a^For approximately how long did your most recent HAE attack stop you from performing your normal activities? Median duration for less common sites: “other” (including joints and low back) = <2 h (n = 7); face = <2 to 2-4 h (n = 6); respiratory/laryngeal = 4-12 h (n = 3). Differences across sites not statistically significant (p = 0.560).

**Figure 3:**
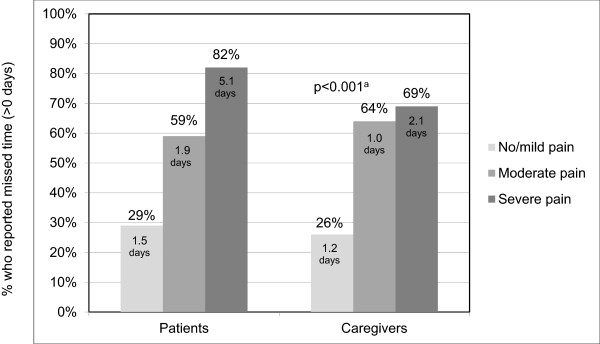
**Work/school absenteeism during last attack by pain severity.** Percentage who missed time and reported mean days missed. ^a^p-values for difference in percentage who missed time. Percentages based on 72 patients who were employed or in school and provided absenteeism data. Percent of caregivers based on full sample (N = 164). Note: Means based on number of patients (n = 40) and caregivers (n = 86) who missed time. Caregiver time includes leisure time. Data missing for missed time for 31 caregivers.

Of the 164 patients, 120 (73%) reported that they were either working or going to school (73 working full-time; 21 working part-time; 7 in school; 19 both working and in school), and 72 (60%) provided work/school absenteeism data, which was optional in the survey. Of these 72 patients, 40 (56%) reported missing time from work/school during the last attack. Higher pain severity of the last attack was associated with a greater impact on work/school productivity (r = 0.39; p = 0.001). On a scale from 0–10 (higher worse), the last attack impaired patients’ work/school productivity a mean of 4.4. The last attack prevented patients from their normal activities an average of 4–12 hours and this did not vary significantly by body site (Figure 2).Figure 3 shows that work/school absenteeism for patients and caregivers during the last attack increased as pain severity increased. Overall, 86 patients (52%) reported that they had assistance from a caregiver during the last attack (primarily a family member), including 69% (n = 25) of patients with severe pain, 64% (n = 47) of patients with moderate pain, and 26% (n = 14) of patients with no pain or mild pain (p < 0.001). Patients with severe pain also reported that their caregivers missed more time during the last attack than patients with no pain/mild pain or moderate pain (2.1 vs. 1.2 and 1 day, respectively; p = 0.015).

#### *Impact of HAE on productivity in between attacks for patients and caregivers*

In addition, absenteeism between attacks over the past six months for both patients and caregivers increased as attack frequency increased. Specifically, the average numbers of missed days for patients were 2.2, 6.0, and 11.6 among patients with attacks less than once a month, at least once a month but less than once a week, and at least once a week, respectively (p = 0.128), and they reported that their caregivers missed 3.3, 3.6, and 10.1 days, respectively (p = 0.088). Patients also experienced decreased productivity due to HAE between attacks, with a mean of 1.9 on a 0–10 scale.

#### *Total estimated absenteeism over 12 months*

Based on estimated attack frequency and reported absenteeism data, overall, patients are estimated to miss approximately 20 days of work/school per year due to HAE. Patients who reported severe pain during the recent attack had a higher absenteeism estimate of approximately 28 days per year (Table 2).

**Table 2:** Reported and estimated work/school absenteeism due to HAE

**Pain severity of the last attack**	**Estimated attacks per year**	**Reported time missed for last attack (days)**	**Reported time missed between attacks in past 6 months (days)**	**Estimated total absenteeism over 12 months**^**a **^**(days)**
	**Mean ± SD / median (range) (95% CI)**	**Mean ± SD / median (range) (95% CI)**	**Mean ± SD / median (range) (95% CI)**	**Mean ± SD / median (range) (95% CI)**
*Overall (n = 67)*	*33.2 ± 39.6*	*1.0 ± 1.6*	*1.9 ± 2.5*	*19.9 ± 35.0*
	*12.0 (1.0-156.0)*	*0.25 (0.0-8.0)*	*1.0 (0.0-11.0)*	*8.0 (0.0-210.0)*
	*(23.7-42.7)*	*(0.6-1.4)*	*(1.3-2.5)*	*(11.5-28.2)*
None/mild (n = 20)	43.7 ± 48.7	0.5 ± 0.9	2.1 ± 2.7	19.3 ± 47.0
	24.0 (1.0-156.0)	0.0 (0.0-3.0)	1.1 (0.0-11.0)	5.0 (0.0-210.0)
	(32.8-54.6)	(0.3-0.7)	(0.5-1.7)	(8.8-29.8)
Moderate (n = 33)	30.4 ± 37.3	1.0 ± 1.6	1.2 ± 1.6	16.8 ± 29.2
	12.0 (1.0-156.0)	0.4 (0.0-8.0)	0.5 (0.0-6.0)	6.3 (0.0-140.0)
	(6.3-17.7)	(0.75-1.2)	(1.0-1.4)	(11.8-20.8)
Severe (n = 14)	24.8 ± 28.1	1.8 ± 1.9	3.3 ± 3.4	28.2 ± 28.2
	12.0 (5.0-104.0)	1.1 (0.0-7.0)	3.0 (0.0-10.0)	17.3 (0.0-104.0)
	(17.3-32.3)	(1.3-2.3)	(2.4-4.2)	(20.7-35.7)

Note: Estimates based on patients who provided absenteeism data (n = 72); 5 outliers identified and removed (estimated attacks per year ≥ 208 or estimated absenteeism ≥ 258 days); thus, final sample size = 67.

^a^Estimated absenteeism over 12 months calculated as the mean of the estimates computed at the individual-level; given that the absenteeism data are skewed, these estimates differ from those based on the mean time missed as a group.

Confidence Intervals of means reported.

#### *Long-term consequences for education and careers*

**Figure 4:**
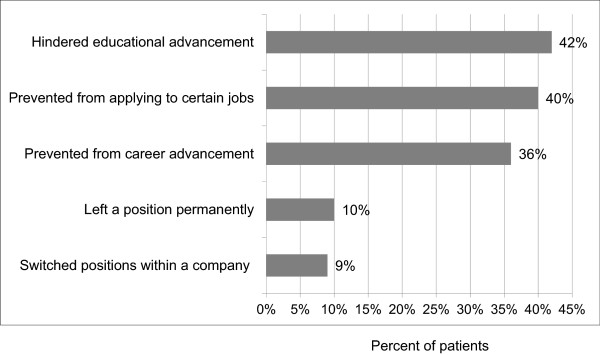
**Impact of HAE on career and education.** Note: 84 patients (51%) reported that HAE hindered their educational and/or career advancement.

High proportions of patients reported that HAE has hindered their career and/or educational advancement, prevented them from applying to certain jobs, caused them to leave a position permanently, or caused them to switch positions within a company (Figure 4). This was significantly related to attack frequency; 57% (n = 60) of patients with attacks at least once a month reported that HAE has hindered their career and/or educational advancement compared to 41% (n = 24) of patients with attacks less than once a month (p = 0.043).

## Discussion

This study describes the socioeconomic burden of HAE in Europe, related to the treatment of acute attacks and routine care, work/school absenteeism and productivity impairment during and in between attacks, and long-term consequences for career and educational achievement. The findings suggest that HAE may lead to substantial direct and indirect costs, corroborating results observed in the US [21].

Attack pain severity was an important predictor of direct and indirect resource utilization. Greater pain severity was associated with an increased likelihood of using the emergency department for treatment of the attack, treating the attack with an acute medication, decreased productivity during the attack, greater absenteeism for patients and their caregivers during the attack, and greater six-month medical resource utilization. Similar to our findings, Nordenfelt *et al*. also show in Sweha-Reg that absenteeism correlates with increasing patient reported attack severity [25].

Time lost from usual activities did not vary significantly by body site affected, suggesting that attacks in all body sites have a limiting effect. Attack frequency was also associated with socioeconomic burden. Over the past six months, higher attack frequency was associated with decreased productivity and higher absenteeism. Higher attack frequency was also associated with difficulty advancing in one’s education or career. Such long-term productivity losses represent a substantial opportunity cost for the individual and their family related to not achieving their full potential in their education and careers.

As with any study of this nature, while systematic recruitment was attempted, participation was ultimately based on self-selection, which may affect the representativeness of the study population. Although the estimated total number of HAE patients differs largely between the countries involved, equal numbers were attempted to recruit in each country, so as to receive the target sample size of 150 patients, which is consistent with other burden of illness studies in rare diseases [26,27] Attempts were made to ensure generalizable country specific sampling by ways of systematic or random selection. Yet, it cannot be excluded that the willingness of a patient to participate in a survey, increases with disease severity.

We also performed an analysis of a potential selection bias by route of recruitment, either via patient organization or clinical center. Such analysis demonstrated no significant difference in the proportion of patients recruited via each route of recruitment. Also, the clinical characteristics of the recent attack (pain severity, swelling severity, time to treatment in each country, as well as duration of swelling in German and Spanish participants) showed no significant differences. Outcome measures for direct resource utilization (percentage of patients with medication to treat attack at home, percentage of treated attacks, visits for treatment of recent attack, medication used to treat anxiety or depression) were also not significantly different between patients by recruitment route and country. Likewise, indirect resource utilization as measured by impact items (impact on work and school productivity as well as impact on daily activites, each with recent attack or between attacks) and report on being prevented from career or educational advancement were not significantly different between patients by recruitment route and country. Despite different routes of recruitment, the data obtained seem to be consistent.

The survey relied on self-reported, retrospective data, which carries the potential for recall bias, although HAE patients often keep symptom diaries. Data collected reflect the treatment options available at the time of the study (in 2011), which varied between countries. As this study was observational with small numbers representing different treatments, treatment-specific subgroup analyses were not feasible. For the most part, country comparisons were not feasible given different treatment availability, cultural attitudes toward health service use, and health care system configuration; all country comparisons made, including those regarding attack frequency and medication use, may reflect such differences. In estimating absenteeism over a one year period, it was assumed that the severity of the last attack and absenteeism during the last attack were representative of the average attack for the patient, although the severity of attacks is known to be unpredictable. In addition, absenteeism data were only available for 60% (n = 72) of patients either working or attending school [56% (n = 40) of whom reported absenteeism], although attack severity and frequency did not differ for these patients compared to patients for whom such data was unavailable. Also, this study did not assess lost leisure time for patients and therefore may underestimate patient-level burden. Nevertheless, our findings, particularly those related to absenteeism and long-term career/educational impact, are highly comparable to those observed in the US HAE burden of illness and Sweha-Reg studies [21,25,28]. Moreover, this study adds to the literature in the assessment of patient-reported use of caregivers due to HAE, which contributes to patient-level and societal burden.

## Conclusion

As HAE is a rare disease, economic burden at the population level may be relatively small; however, our findings highlight the substantial socioeconomic burden HAE poses to patients and their families. Our data show that patients with frequent and severe attacks experience greater interference in their education and careers, as well as increased use of emergency departments and acute medication. As such, further exploration is warranted with respect to the potential benefits of different treatment strategies.

## Abbreviations

HAE: Hereditary angioedema; HAE-BOIS-Europe: Hereditary Angioedema Burden of Illness Study in Europe.

## Competing interests

The study was funded by ViroPharma SPRL-BVBA.

^1^EAP has received sponsorship for educational purposes and has provided consultancy services or has participated in clinical trials sponsored by CSL-Behring, Jerini AG/Shire, Sobi, and ViroPharma.

^2^AB has been involved in clinical research or educational events involving CSL Behring, Jerini AG/Shire, Sobi and ViroPharma.

^3^ KB and EH worked for Oxford Outcomes Inc., an ICON plc company, at the time this study was undertaken. Oxford Outcomes Inc. consults for ViroPharma.

^4^ZS is an employee of ViroPharma.

^5^SW receives consulting fees from ViroPharma.

^6^HBB is the Executive Director of HAEi – International Patient Organization for C1 Inhibitor Deficiencies, which receives funding from most pharmaceutical companies, including ViroPharma.

^7^TC received sponsorship for educational purposes, has been paid for providing consultancy services, or has taken part in clinical trials sponsored by Jerini AG/Shire, CSL-Behring, Pharming NV, Sobi, and ViroPharma.

## Authors’ contributions

EAP, AB and TC contributed to the study design, participated in the acquisition and analysis of patient data and provided substantive input to the manuscript. KB and EH contributed to the study design, provided input on survey design, analysis, and medical writing support on behalf of the BOIS-EU Steering Committee. ZS coordinated the work of the Steering Committee, performed literature searches, and provided substantive input to the manuscript. SW contributed to the study design and drafted the manuscript. HBB contributed to the study design, participated in the acquisition of patient data and provided substantive input to the manuscript. All authors read and approved the final manuscript.
